# Continuous expression of CD83 on activated human CD4^+^ T cells is correlated with their differentiation into induced regulatory T cells

**DOI:** 10.3892/mmr.2015.3796

**Published:** 2015-05-18

**Authors:** LIWEN CHEN, SHIHE GUAN, QIANG ZHOU, SHOUQIN SHENG, FEI ZHONG, QIN WANG

**Affiliations:** 1 Departments of Laboratory Medicine, The Second Hospital of Anhui Medical University, Hefei, Anhui 230601, P.R. China; 2 Medical Research Center, The Second Hospital of Anhui Medical University, Hefei, Anhui 230601, P.R. China; 3 Department of Medical Oncology, The First Hospital of Anhui Medical University, Hefei, Anhui 230022, P.R. China

**Keywords:** CD83, differentiation, activation, co-localization, regulation

## Abstract

CD83 is a widely recognized surface marker for mature dendritic cells, which are essential for priming naïve CD4^+^ T cells into effector cells. However, CD83 is also expressed on activated CD4^+^ T cells, which remains an enigma in T-cell mediated immunity. Therefore, the identification of the biological features and regulation of the expression of CD83 on activated CD4^+^ T cells is important in understanding the function of CD83 in the adaptive immune response. The present study revealed a time-dependent manner of the expression of CD83 on anti-CD3/CD28-stimulated human CD4^+^ T cells, which is characterized by the maximum expression at day 2 and a significant decrease at day 3. The reduced expression is not a result of a reduced rate of cell proliferation. The activation of interleukin-2 and secretion of interferon-γ accumulated progressively from day 1 to 3. Of note, sustained expression of CD83 was observed when CD4^+^ T cells were induced by transforming growth factor-β to differentiate into CD4^+^CD25^+^ forkhead box P3^+^ regulatory T (iTreg) cells. Confocal immunofluorescence microscopy analysis demonstrated that CD83 was highly co-localized with CD25 on activated CD4^+^ T cells. In conclusion, the findings of the present study suggested that the continuous expression of CD83 on activated human CD4^+^ T cells is correlated with their differentiation into iTreg cells.

## Introduction

CD83 is a type I transmembrane glycoprotein with a highly glycosylated N-terminal ectodomain and a short C-terminal intracellular domain. Being absent from the majority of resting cells, CD83 is predominantly induced on the surface of mature dendritic cells (mDCs) and activated T and B lymphocytes (1–3). Increasing evidence has demonstrated the significant regulatory roles of CD83 in the central and peripheral immune system (4–14). CD83 was demonstrated to be essential for the lineage commitment of CD4^+^ T cells in the thymus (4). A previous study has demonstrated that membrane CD83 (mCD83) promotes the expression of major histocompatability complex (MHC) class II and CD86 on mDCs by inhibiting membrane-associated RING-CH1 (MARCH1)-dependent ubiquitination and degradation of the two target molecules (5). The co-stimulatory effects of mCD83 on mDCs per se for CD4^+^ T cells have remained controversial (4,6–8). However, soluble CD83 (sCD83), which is produced predominantly by ectodomain shedding, has clear suppressive effects *in vitro* and *in vivo* (9–14). A previous study by our group demonstrated that sCD83 suppresses T-cell proliferation and the secretion of interleukin (IL)-2 and interferon (IFN)-γ through prostaglandin E2 (PGE_2_) produced by monocytes (15).

A previous study demonstrated that native or forced expression of CD83 confers an immunosuppressive function to CD4^+^ T cells (16). However, a previous study using short hairpin (sh)RNA-mediated gene silencing of CD83 on CD4^+^ T cells revealed a reduced proliferation and lower production of IL-2 and IL-17 by the CD4^+^ T cells, indicating that CD83 serves as a positive co-stimulator for CD4^+^ T cells (17). It was noted that genetic manipulation may cause unintended effects to the target cells. On the other hand, modified expression of CD83 is likely paralleled by concurrent changes of co-stimulatory molecules on CD4^+^ T cells, since CD83 is an important regulator of MHC class II and the expression of CD86 (5). Therefore, the biological and functional definition of the expression of CD83 on CD4^+^ T cells remains to be elucidated.

In the present study, the expression of CD83 on CD4^+^ T cells was assessed. The effects of stimulation with a (TGF)-β on the expression of CD83 on CD4^+^ T cells as well as on their differentiation into CD4^+^CD25^+^ forkhead box (Fox) P3^+^-induced regulatory T (iTreg) cells were investigated.

## Materials and methods

### Lymphocyte purification and cell culture

Usin Ficoll-Hypaque density gradient centrifugation at 900 × mononuclear cells were isolated from the blood of healthy donors who provided written informed consent. This study was approved by the Ethics Committee of the Second Hispital of Anhui Medical University (Hefei, China). The mononuclear cell suspension (8 ml) was added into a T-25 culture flask and incubated at 37°C with 5% CO_2_ for 2 h. The cells were gently agitated, the non-adherent cells were aspirated, and adherent monocytes and B cells were discarded. Alternatively, untouched CD4^+^ T cells were purified from mononuclear cells using a CD4^+^ T-cell isolation kit (cat. no. 130-096-533) and magnetic columns (cat. no. 130-042-306) (both from Miltenyi Biotech, Bergisch Gladbach, Germany). This procedure routinely provided >95% pure CD4^+^ T cells. Non-adherent lymphocytes or purified CD4^+^ cells were cultured in RPMI-1640 containing 10% fetal bovine serum, 10 mM 4-(2-hydroxyethyl)-1-piperazineethanesulfonic acid and 1% penicillin-streptomycin (all purchased from Invitrogen Life Technologies, Carlsbad, CA, USA) at 1×10^6^ cells/ml in 24-well plates, in the presence of pre-coated agonistic murine anti human CD3 monoclonal antibody (mAb; clone UCHT-1; cat. no. 555329; 0.5 *µ*g/ml) and soluble agonistic murine anti human CD28 mAb (clone ANC28.1/5D10; cat. no. 177–820; 1.0 *µ*g/ml), purchased from BD Biosciences, Franklin Lakes, NJ, USA and Ancell, Bayport, MN, USA, respectively. To assess the importance of TGF-β regulation on the expression of CD83 on CD4^+^ T cells, a final concentration of 2 ng/ml exogenous TGF-β (Sigma-Aldrich, St. Louis, MO, USA) was added to the cultures at day 0. All cells were harvested 1, 2 or 3 days following incubation for flow cytometry and confocal immunofluorescence microscopy analysis.

### In vitro cell proliferation assay and cytokine detection

As mentioned above, purified CD4^+^ T cells were collected and stimulated with agonistic anti-CD3/CD28 at 10^5^/ml in 96-well plates. Determination of cell proliferation was performed 1, 2 and 3 days later using a Cell Counting kit-8 (Dojindo, Kumamoto, Japan), according to the manufacturer's instructions. Cell viability was determined by measuring the absorbance at 450 nm using a multiwell scanning spectrophotometer (KHB ST-360; Kehua Bio-Engineering, Shanghai, China). The experiments were repeated in triplicate wells. The levels of IL-2 and IFN-γ in CD4^+^ T-cell culture supernatants were measured in duplicate for each of the serial aliquots using a Human IL-2 Quantikine ELISA kit (cat. no. D2050) and Human IFN-γ Quantikine ELISA kit (cat. no. DIF50), purchased from R&D Systems (Minneapolis, MN, USA), according to the manufacturer's instructions.

### Flow cytometric analysis

Non-adherent lymphocytes or purified CD4^+^ T cells were collected at 0, 1, 2 and 3 days following stimulation, and the cells were stained with phycoerythrin (PE)-conjugated anti-CD83, fluorescein isothiocyanate (FITC)-conjugated anti-CD4 or FITC-conjugated anti-CD25 (All from BioLegend, San Diego, CA, USA) and incubated on ice for 30 min in the dark. The controls were prepared using FITC- or PE-conjugated isotype controls. The intracellular staining of TGF-β-treated CD4^+^ T cells with anti-Foxp3 mAb was performed using an anti-human Foxp3 staining set (eBioscience, San Diego, CA, USA) according to the manufacturer's instructions. The cells were subsequently analyzed using a flow cytometer (Beckman Coulter Epics XL; Beckman Coulter, Miami, FL, USA) by using a two-color acquisition method and the data were analyzed by using FlowJo 7.6.1 software (Treestar, Inc., Ashland, OR, USA).

### Confocal immunofluorescence microscopy

Following stimulation with anti-CD3/CD28 for 2 days, purified CD4^+^ T cells were washed twice with ice-cold phosphate-buffered saline (PBS) and fixed in 4% (w/v) paraformaldehyde (Sigma-Aldrich) in PBS for 15 min at room temperature (20°C). The cells were subsequently stained with FITC-conjugated anti-CD25 mAb and PE-conjugated anti-CD83 mAb, and incubated on ice for 30 min in the dark. Fluorescently-labeled cells were observed and recorded by an examiner, in a blinded manner, using a Zeiss LSM 410 inverted laser scan microscope (Carl Zeiss, Oberkochen, Germany) equipped with Ar488, Kr568 and HeNe633 lasers. Non-specific fluorescence was assessed by incubating cells with FITC- or PE-conjugated isotype controls.

### Statistical analysis

Values presented in the figures are representative of at least three independent experiments and are expressed as the mean± standard derivation. Comparisons between the expression levels of CD83 at days 1, 2 and 3 were determined by Student's t-test with SPSS 13.0 software (SPSS, Inc., Chicago, IL, USA). P<0.05 was considered to indicate a statistically significant difference.

## Results

### Time-dependent expression of CD83 on CD4^+^ T cells is stimulated by anti-CD3/CD28

**Figure 1:**
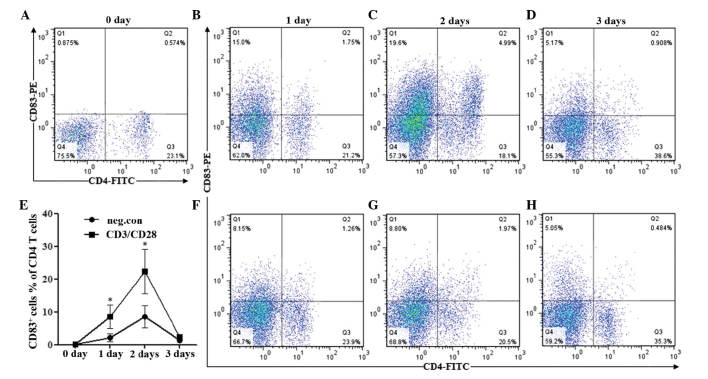
Expression of CD83 on non-adherent lymphocytes. (A) Freshly isolated (0 d) non-adherent lymphocytes were directly stained with FITC-labeled CD4 and PE-labeled CD83 and analyzed by flow cytometry. (B–D) Alternatively, non-adherent lymphocytes were stimulated with anti-CD3/CD28 or (F–H) left unstimulated for 1–3 days. The cells were subsequently stained with FITC-labeled anti-CD4 and PE-labeled anti-CD83, and analyzed by flow cytometry. Dot plots are representative of three independent experiments. (E) Values are expressed as the mean ± standard deviation of the percentages of CD83-positive cells in total CD4^+^ T cells of three independent experiments (^*^P<0.05, compared with the unstimulated group on the same day). FITC, fluorescein isothiocyanate; PE, phycoerythrin; CD, cluster of differentiation; neg.con, negative control.

To assess the time-dependent kinetics of the expression of CD83 on CD4^+^ T cells, non-adherent lymphocytes isolated from mononuclear cells were stimulated with agonistic anti-CD3/CD28 and the expression of CD83 on CD4^+^ T cells was determined by flow cytometry 1, 2 and 3 days following stimulation. As shown in Fig. 1A, CD83 was rarely expressed on resting CD4^+^ T cells (0 days), whereas the percentages of CD83-positive cells in the total CD4^+^ T cells were significantly upregulated at day 1 (7.69%) and markedly increased to 21.61% by day 2 (Fig. 1B and C). However, the activation-induced expression of CD83 on CD4^+^ T cells decreased significantly to 2.30% at day 3 (Fig. 1D). By contrast, unstimulated CD4^+^ T cells demonstrated less severe increases in the expression of CD83 compared with those in the stimulated group on days 1 and 2, and on day 3, CD83 were similar to those in the stimulated group (Fig. 1E–H). The differences in the percentage of CD83^+^ cells (with regard to the total CD4^+^ T-cell population) between the anti-CD3/CD28-stimulated and the unstimulated group were statistically significant (P<0.05) at days 1 and 2 (Fig. 1E).

**Figure 2:**
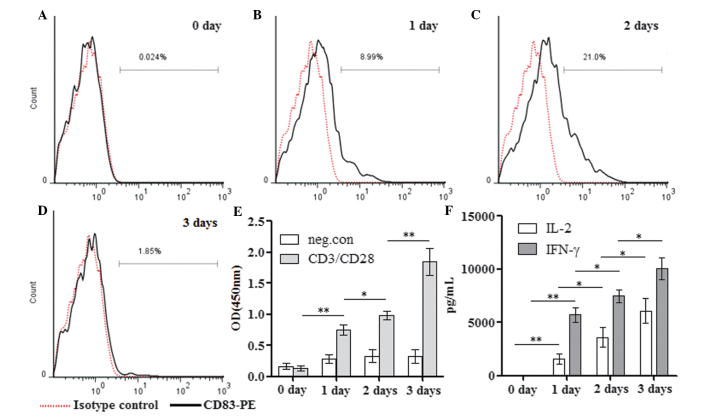
Time-dependent expression of CD83, proliferation and the production of IL-2 and IFN-γ in CD4^+^ T cells. Following purity identification, the purified CD4^+^ T cells were either (A) directly stained with PE-labeled CD83 or (B–D) stimulated with anti-CD3/CD28 for 1–3 days, followed by staining with PE-labeled CD83. All samples were analyzed by flow cytometry and the typical flow cytometric histograms indicating the percentages of CD83-positive cells are shown. (E) Additionally, cell proliferation at days 1, 2 and 3 were determined using Cell Counting Kit 8. (F) The levels of IL-2 and IFN-γ in the supernatants from 1-, 2- and 3-day cultures of activated CD4^+^ T cells were analyzed by ELISA. Values are expressed as the mean ± standard deviation of three independent experiments (^**^P<0.01; ^*^P<0.05). PE, phycoerythrin; CD, cluster of differentiation; IFN, interferon; IL, interleukin; neg.con, negative control.

Purified CD4^+^ T cells were used to confirm the time-dependent surface expression of CD83. As shown in Fig. 2A–D, anti-CD3/CD28 stimulation of CD4^+^ T cells led to an upregulation of CD83 on days 1 and 2 and substantially low levels of CD83 on CD4^+^ T cells on day 3. The time-dependent kinetics were consistent with those observed in non-adherent lymphocytes. These data suggested that CD83 was significantly upregulated; however, it was transiently presented on CD4^+^ T cells activated by the canonical CD3/CD28 signal.

### Decreased expression of CD83 is not caused by a reduced proliferation or activation of CD4^+^ T cells

The present study next determined whether the decreased expression of CD83 on day 3 was a result of reduced proliferation or activation of target CD4^+^ T cells. Purified CD4^+^ T cells were stimulated with anti-CD3/CD28 for 1, 2 and 3 days, and cell proliferation as well as the levels of IL-2 and IFN-γ in culture supernatants were analyzed. This experiment paralleled the detection of the expression of CD83 on CD4^+^ T cells as mentioned above. By contrast to fluctuated surface expression of CD83, the proliferation of CD4^+^ T cells stimulated by anti-CD3/CD28 was continuously increased from days 1 to 3 (Fig. 2E). Similarly, progressively increased secretion of IL-2 and IFN-γ was observed in CD4^+^ T cells (Fig. 2F). Therefore, the notable decrease in the surface expression of CD83 at day 3 was not a result of reduced proliferation or the activation of CD4^+^ T cells.

### Co-localization of CD83 and CD25 on activated CD4^+^ T cells

**Figure 3:**
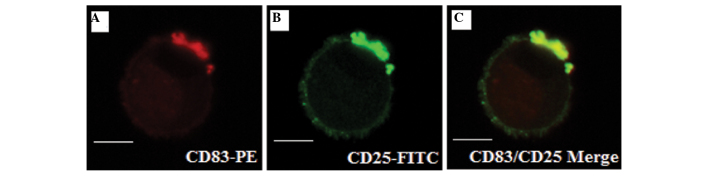
Surface co-localization of CD83 and CD25 on activated CD4^+^ T cells. Purified CD4^+^ T cells were stimulated with anti-CD3/CD28 for 2 days, deposited on slides, stained with (A) PE-labeled anti-CD83 and (B) FITC-labeled anti-CD25 and visualized by confocal microscopy. (C) The images of PE-labeled anti-CD83 and FITC-labeled anti-CD25 were merged to reveal an overlap of the two proteins. Images were captured from a x63 objective of a Zeiss LSM 410 microscope. Images are representative of three separate experiments (Scale bar, 5 *µ*M). FITC, fluorescein isothiocyanate; PE, phycoerythrin; CD, cluster of differentiation.

Previous studies have reported that the mRNA expression of CD83 was predominantly in the naturally occurring CD4^+^CD25^+^ T (nTreg) cells, which rapidly expressed large quantities of surface CD83 upon activation (16). It was therefore of interest to determine whether CD83 is co-localized with CD25 on activated CD4^+^ T cells. Purified CD4^+^ T cells were stimulated with anti-CD3/CD28 for 2 days, double-labeled with CD25-FITC/CD83-PE and observed by immunofluorescence microscopy. The results demonstrated that CD83 and CD25 were accumulated on the surface of activated CD4^+^ T cells (Fig. 3A and B). Furthermore, merging of the two images revealed a high degree of co-localization between CD83 and CD25 on the surface of the activated CD4^+^ T cells (Fig. 3C). These results suggested that there was a significant correlation between the expression levels of CD83 and CD25 on anti-CD3/CD28-stimulated CD4^+^ T cells.

### TGF-β restores the expression of CD83 on CD4^+^ T cells

**Figure 4:**
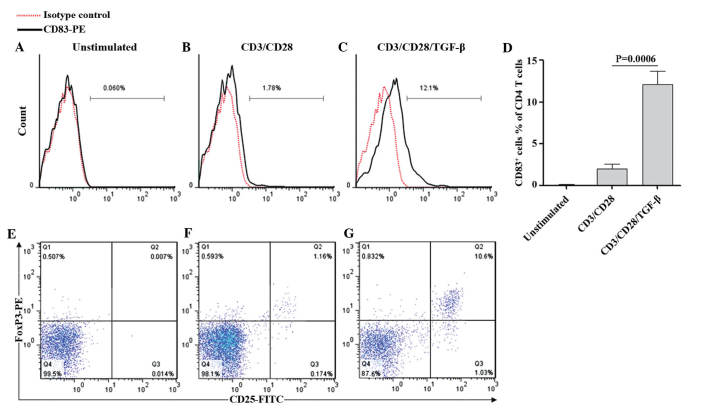
TGF-β restored the expression of CD83 in CD4^+^ T cells. Purified human CD4^+^ T cells were (A) left unstimulated or (B and C) stimulated with anti-CD3/CD28 in the presence or absence of TGF-β. Following 3 days of incubation, all samples were collected, stained with PE-labeled anti-CD83 and analyzed by flow cytometry. The typical flow cytometric histograms indicating the percentages of CD83-positive cells are shown in A, B and C. (D) Quantification of the number of CD83-positive cells in the total CD4^+^ T cell population. Values are expressed as the mean ± standard deviation of four independent experiments and P-values were calculated by Student's t-test. Additionally, the (E) unstimulated, (F) anti-CD3/CD28- and (G) anti-CD3/CD28/TGF-β-treated CD4^+^ T cells were stained with FITC-labeled anti-CD25, followed by intracellular FoxP3-PE staining and analyzed by flow cytometry. The data demonstrated representative data for the donors in A–C and E–G. FITC, fluorescein isothiocyanate; PE, phycoerythrin; CD, cluster of differentiation, TGF, transforming growth factor; Fox, forkhead box.

Induced CD4^+^CD25^+^FoxP3^+^ T cells represent an important sub-type of CD4^+^ T cells, termed iTreg cells, which are instrumental in the maintenance of immunological tolerance (18). The highly co-localized expression of CD83 and CD25 on CD4^+^ T cells suggests that the expression of CD83 is closely associated with the differentiation of CD4^+^ T cells into iTreg cells upon activation. The present study aimed to determine whether the sudden decrease in the expression of CD83 on day 3 was due to the differentiation of CD4^+^ T cells, since the canonical CD3/CD28 signal rarely drives the generation of iTreg cells. With this suggestive evidence, purified CD4^+^ T cells were stimulated with anti-CD3/CD28 in the presence or absence of TGF-β, an immunosuppressive cytokine, which controls the balance between iTreg cells and pathogenic effector T cells (19). The expression of CD83 on CD4^+^ T cells was detected 3 days later using flow cytometry. In addition, the differentiation of CD4^+^ T cells triggered by TGF-β signaling was identified by co-expression analysis of FoxP3 and CD25. The experiments demonstrated that TGF-β signaling increased the percentage of CD83-postive cells in total CD4^+^ T cells to 12.1% on day 3, while stimulation without TGF-β only resulted in 1.78% CD38-positive cells (Fig. 4A–C). The difference was statistically significant according to Student's t-test (P=0.0006; Fig. 4D). Furthermore, it was observed that, in contrast to anti-CD3/CD28 stimulation, the combination with TGF-β induced a significant increase in the co-expression of FoxP3 and CD25 on CD4^+^ T cells on day 3 (Fig. 4E–G). In conclusion, these findings demonstrated that the decreased surface expression of CD83 on activated CD4^+^ T cells can be restored by TGF-β, which simultaneously drives the differentiation of CD4^+^ T cells into iTreg cells.

## Discussion

A growing body of *in vitro* and *in vivo* evidence has demonstrated an immunosuppressive role of sCD83 in T cell-mediated immunity (9–14). However, the effect of mCD83 expressed on antigen-presenting cells (APCs), including dendritic cells and B lymphocytes, remains a matter of controversy (4,6–8). Similarly, CD83 expressed on the surface of CD4^+^ T cells poses a novel challenge to elucidate the biological and functional behavior of mCD83. The present study demonstrated that CD83 was expressed in a time-dependent manner on activated human CD4^+^ T cells, which reached the maximum at day 2 and decreased significantly on day 3. These time-dependent kinetics of the expression of CD83 are consistent with observations using CD4^+^ T cells isolated from BALB/c mice and stimulated with anti-CD3 and IL-2 in the presence of irradiated CD4-depleted splenocytes as APCs (16). The decreased expression of CD83 at day 3 was not a result of reduced proliferation or activation of CD4^+^ T cells, in that IL-2 and IFN-γ production was sustained until day 3. Therefore, these findings demonstrated the fine-tuning of activation-induced expression of CD83 on CD4^+^ T cells. Of note, non-CD4 cells in non-adherent lymphocytes expressed considerable quantities of surface CD83. It was suggested that this synchronous presentation of CD83 originates from CD8^+^ T cells and remaining B cells upon activation (20,21). On the other hand, unstimulated CD4^+^ T cells also expressed certain quantities of surface CD83, which may be explained by the spontaneous activation of undepleted monocytes in non-adherent lymphocytes, which in turn stimulate and activate CD4^+^ T cells.

Upon T-cell receptor (TCR)-mediated cell activation, naïve CD4^+^ T cells differentiate into at least four major lineages (Th1, Th2, Th17 and iTreg cells), which can be distinguished by their specialized expression profiles, unique cytokine production and effector functions (22). The present study suggested that differentiation of CD4^+^ T cells, which follows cell activation, may be responsible for the downregulation of CD83 on CD4^+^ T cells stimulated by the canonical CD3/CD28 signal. By using a CD83 reporter mouse expressing enhanced green fluorescent protein under the control of the CD83 promoter, a previous study has demonstrated that CD83 was predominantly identified in CD4^+^CD25^+^ and in CD4 memory cells (21). Similarly, an *in vitro* study revealed that the mRNA expression levels of CD83 were differentially expressed in nTreg cells, which rapidly expressed large quantities of surface CD83 upon activation (16). In this context, the present study examined the spatial positions of CD83 and CD25 on activated CD4^+^ T cells by confocal microscopy. Of note, co-localization of CD83 and CD25 on the surface of activated CD4^+^ T cells was observed. CD25 is the α-chain of the IL-2 receptor, and an IL-2 signal is essential for the differentiation, expansion and function of Tregs (23,24). The function of highly co-localized presentation of CD83 and CD25 remains to be elucidated; however, it may be associated with the stabilization of surface CD25 on target cells by CD83. A previous study has demonstrated that the transmembrane domain of CD83 inhibited the activity of membrane-associated ring finger (C3HC4) 1, E3 ubiquitin protein ligase (MARCH1), a member of the MARCH family of membrane-bound E3 ubiquitin ligases, which ubiquitinate and downregulate cell surface molecules (5).

Numerous studies have demonstrated that the presence of TGF-β at the onset of cell cultures can drive naïve CD4^+^ T cells to differentiate into iTreg cells (19,25). The present study therefore investigated the effects of TGF-β regulation on the expression of CD83 on CD4^+^ T cells. The findings support the evidence that the addition of TGF-β can restore the surface expression of CD83 on purified CD4^+^ T cells stimulated with anti-CD3/CD28 for 3 days. In addition, the combined TGF-β stimulation drove CD4^+^ T cells towards a phenotype of CD4^+^CD25^+^FoxP3^+^ iTreg cells. As mentioned above, the close association between CD83 and iTreg cells was also demonstrated to be present in nTreg cells, which develop in the thymus and constitutively express CD25 (16,21). These observations may have important implications for CD83 regulation on Treg cells. Therefore, it is conceivable that the sustained presentation of CD83 on CD4^+^ Treg sub-sets is required for Treg induction, differentiation, survival or functional maintenance. Defining the factors which regulate the expression of CD83 and understanding the mechanisms of CD83 regulation on regulatory T cells may facilitate the development of Treg cells and novel therapeutic strategies in immune intervention.

In conclusion, the results of the present study suggested that CD83 is presented in a time-dependent manner on CD4^+^ T cells stimulated by the canonical CD3/CD28 signal. The maximum expression of CD83 expression was observed at day 2 and was followed by a marked decrease at day 3. Of note, the addition of TGF-β at the onset of stimulation, which drove the differentiation of CD4^+^CD25^+^FoxP3^+^ Tregs, restored the expression of CD83 on day 3. Furthermore, CD83 was highly co-localized with CD25, as observed by fluorescence microscopy. Therefore, the present study suggested that the continuous expression of CD83 on activated human CD4^+^ T cells was correlated with their differentiation into iTreg cells. Establishing the functional connection between the expression of CD83 and iTregs may facilitate the further elucidation of iTregs and their clinical application.
